# Evaluation of the HOOF-Print assay for typing *Brucella abortus *strains isolated from cattle in the United States: results with four performance criteria

**DOI:** 10.1186/1471-2180-5-37

**Published:** 2005-06-23

**Authors:** Betsy J Bricker, Darla R Ewalt

**Affiliations:** 1 Bacterial Diseases of Livestock Research Unit, National Animal Disease Center, Agricultural Research Service, United States Department of Agriculture, 2300 Dayton Rd, Ames, IA, 50010, USA; 2 Diagnostic Bacteriology Laboratory, National Veterinary Services Laboratories, Animal and Plant Health Inspection Service, United States Department of Agriculture, 1800 Dayton Rd, Ames, IA, 50010, USA

## Abstract

**Background:**

A fundamental question that arises during epidemiological investigations of bacterial disease outbreaks is whether the outbreak strain is genetically related to a proposed index strain. Highly discriminating genetic markers for characterizing bacterial strains can help in clarifying the genetic relationships among strains. Under the auspices of the European Society of Clinical Microbiology and Infectious Diseases, the European Study Group for Epidemiological Markers (ESGEM) established guidelines for evaluating the performance of typing systems based of a number of criteria. Recently, HOOF-Print genotype analysis, a new method for typing *Brucella abortus *strains based on hypervariability at eight tandem repeat loci, was described. This paper evaluates the HOOF-Print assay by four of the criteria set out by the ESGEM: typeability, reproducibility, power of discrimination, and concordance with other typing methods.

**Results:**

The HOOF-Print Assay was evaluated with a test population composed of 97 unrelated field isolates and 6 common laboratory strains of *B*. *abortus*. Both typeability and reproducibility of the assay were excellent. Allele diversity and frequency varied widely among the eight loci, ranging from 1 to 13 alleles. The power of discrimination, measured by the Hunter-Gaston discrimination index (HGDI), varied by locus ranging from 0 to 0.89, where a maximal value of 1.0 indicates discrimination of all strains. The HGDI values calculated for subgroups sorted by biovar were similar to the values determined for the whole population. None of the individual loci achieved the recommended HGDI threshold of 0.95, but the HGDI of the composite profiles was 0.99 (93 unique genotypes from 97 field strains evaluated), well above the recommended threshold. By comparison, the HGDI value for biovar typing was 0.61 in a test population biased with disproportionate numbers of the less common biovars. Cluster analysis based on HOOF-Print genotypes assembled the strains into hierarchical groups with no apparent association with the time or location of strain isolation. Likewise, these hierarchical groups were not homogeneous with regard to biotype. In one extreme case, two field isolates with identical fingerprints were identified as different biovars by conventional methods.

**Conclusion:**

The main purpose of this study was to assess the ability of HOOF-Print genotyping to discriminate unrelated field strains of *B*. *abortus*, and whether the assay met established requirements for bacterial strain typing methods. The discriminatory power of the assay was remarkable, considering the genetic homogeneity found among species within the genus. The assay met or exceeded all of the recommended levels for the performance criteria of typeability, reproducibility, and power of discrimination, however some inconsistencies with conventional biovar typing were observed. Nevertheless, the results indicate that with cautious interpretation, multilocus genotyping of polymorphic tandem repeats by HOOF-Print analysis could be a valuable complement to routine epidemiological investigations into localized *B*. *abortus *outbreaks.

## Background

During the epidemiological inquiry into a disease outbreak, investigators try to trace the outbreak strain back to the original source of infection. If a potential index strain is identified, the strains are compared to establish a genetic connection between them. To help in this endeavor, a variety of approaches to characterize and classify disease strains have been developed that exploit genotypic and/or phenotypic markers. However, it can be difficult to determine which approaches are practical and informative for routine investigation of the disease agent of choice. Furthermore, direct comparisons of published methods may not be possible because different types of data are generated. At the request of the European Society of Clinical Microbiology and Infectious Diseases, these questions and others were discussed by a panel of international experts, resulting in the formation of the European Study Group on Epidemiological Markers (ESGEM) in 1994. The result of the meeting was a published set of guidelines for the evaluation of epidemiological typing systems [1]. The guidelines describe the performance criteria that should be considered when evaluating a typing system for widespread use. These criteria include: typeability, reproducibility, stability, discriminatory power, epidemiologic concordance, and typing system concordance.

Recently, a new approach has been developed for genetic typing that exploits the greater than normal amount of polymorphism observed within genomic regions containing short (2 to a few tens of base-pairs), tandemly repeated DNA sequences [2-4]. The accelerated mutation rates associated with the repeated sequences are thought to be due to slip-strand mispairing (SSM) [5] and recombination (most likely in the form of gene conversion arising from double-strand break repair [6]), resulting in rapid micro-evolution within the locus. SSM occurs when DNA polymerase misreads the number of repeats on the template strand, typically causing step-wise mutations (the addition or loss of single repeat units). Recombination mechanisms can cause more dramatic expansions or contractions of repeat strings, especially within the very large repeat strings found in eukaryotes [6]. Each variable number tandem repeat (VNTR) locus mutates independently and at an individual rate determined by a number of factors including: the repeat sequence, the size and number of repeat units, the flanking sequence, DNA secondary structure and sequence function [7]. Characterization of these continuously evolving targets has facilitated differentiation of bacterial strains [8,9]. Examination of multiple tandem repeat loci enhances subtype characterization in two ways: firstly, the capacity for genetic discrimination increases when multiple VNTR loci are examined; secondly, the effects of homoplasy (identical alleles arising independently through convergence, reversal or parallelism) may be diminished. Multilocus VNTR analysis (MLVA) has become an effective technique that can discriminate many difficult-to-type bacteria, including many human pathogens such as *Haemophilus influenzae *[10]; *Bacillus anthracis *[11]; *Yersinia pestis *[12]; *Francisella tularensis *[13] and *Mycobacterium tuberculosis *[14].

Brucellosis is an economically important zoonotic disease found throughout many regions of the world, and until recently, throughout the U.S. One causative agent, *Brucella abortus*, is predominantly pathogenic for its natural hosts, cattle and bison (Bovidae family); but it is also pathogenic for humans and several incidental animal species including elk. The disease causes reproductive failure in the host species and chronic health problems in humans.

*B*. *abortus *is a member of a highly homogenous genus, exhibiting ~98.5% nucleotide sequence homology among species [15]. Conventional subtyping of *Brucella *strains into biovars, for epidemiological trace-back, relies on a large array of tests including phage susceptibility, metabolic, biochemical, and serological characterization [16]. Often the differences are subtle. Few biovars are recognized within most *Brucella *species and some species cannot be subtyped at all. Worldwide, *B*. *abortus *has seven biovars; only biovars 1, 2 and 4 occur in the U.S. Differences among these three biovars are minor; discrimination is based on serology and the ability to grow in culture media with certain dyes [16]. Historically, about 85% of U.S. strains were typed as biovar-1.

Previously, we reported the discovery of a reiterated 8-bp sequence, arrayed in tandemly ordered strings [17], that is present in at least eight loci within the sequenced genomes of three *Brucella *species [15,18,19]. A protocol was developed for assessing the number of repeats at each of the eight loci by PCR amplification. The resulting amplicons, containing the entire array of tandem repeats and a small amount of flanking sequence, are sized and the number of repeats is deduced from the length. Since all eight loci have the same 8-bp repeat sequence, the technique was named "HOOF-Prints", an acronym for hypervariable octameric oligonucleotide fingerprints[17].

This paper evaluates the HOOF-Prints technique as it is applied to a diverse collection of *B*. *abortus *field strains representing all three biovar subtypes isolated from throughout the U.S. Our primary goal was to assess how well HOOF-Print genotyping can discriminate among unrelated field strains. Genotyping performance is compared to conventional subtyping into biovars. Individual fingerprint patterns and allelic diversity are presented. The HOOF-Prints technique is also assessed by four of the performance criteria recommended by the ESGEM: typeability, reproducibility, power of discrimination, and concordance with other typing methods. Recommendations for test population criteria are also addressed.

## Results and discussion

### Selection of the test group

An important consideration for evaluating an epidemiological typing system is the selection of a suitable test population. The test population should be large (*N *> 100), consisting of a diverse collection of unrelated strains that is representative of the natural population in which the test is intended to be used [1]. The test group assembled for this study is listed in tabular form in Additional file 1. Information about biovar type, herd location and year of isolation is also included.

All of the strains tested, except for the reference strains, were randomly chosen from diagnostic specimens that had been cultured, positively identified as *B*. *abortus*, biovar typed, and archived by the diagnostic laboratory at the National Veterinary Services Laboratories (APHIS, USDA). As suggested by the ESGEM, the test population was diverse based on the location and date of collection. The field strains originated from 97 different cattle herds, including both dairy and market cattle. Most of the isolates (n = 95) were from the US, but one isolate from Mexico and one isolate from El Salvador were also included. Over half of the isolates (n = 54) were collected in 1991 when brucellosis was still widely disseminated over the country, but on the decline due to the success of the national brucellosis eradication program. In subsequent years, there was a dramatic decrease in outbreaks, so fewer isolates were available. Currently, reports of new outbreaks in the U.S. are uncommon. The primary source of new outbreaks has shifted from domestic cattle to wildlife reservoirs.

To measure the assay's power of discrimination, only one isolate from each herd was included in the test group to prevent statistical over-representation of genotypes. When possible, the infected herds originated from different cities, and in all cases the herds had different owners. However, information regarding possible epidemiological links among the herds was unavailable. A disproportionate number of *B*. *abortus *biovar-2 and biovar-4 isolates were selected to assess the level of natural genotypic diversity within these subtypes. These two biovars are relatively more common in wildlife reservoirs and have recently been on the increase in cattle, due to more cattle outbreaks originating from wildlife. Six common laboratory strains of *B*. *abortus *including the reference strains of *B*. *abortus *biovars 1, 2, and 4, and the two major vaccine strains were also included in this study. Only *B*. *abortus *biovars 1, 2 and 4 were included in the test group, since these are the biovars that occur naturally in the United States.

### HOOF-Print analysis

Eight loci containing octameric tandem repeats were characterized for each of the 103 isolates in the test population. Tandem repeat loci were amplified by PCR with primers directed to the conserved sequences flanking the repeat regions and the total number of repeat units at each locus was deduced from the size of the corresponding amplicon [17]. The results are presented in Additional file 1. The HOOF-Print profiles of some of the strains used in this study have been previously reported in earlier studies (see Additional file 1) [17,20].

At each locus, the alleles are named for the calculated number of repeat units at that locus, such that Allele-4 contains 4 repeat units and Allele-8 contains 8 repeat units. The HOOF-Print (genotypic fingerprint) for an isolate was generated from the allelic profile at all eight loci of that isolate.

### Typeability

An important feature of any typing method is the ability of that method to conclusively classify every sample to a specific type defined by the test parameters. The ESGEM recommends that "*T*" be as close to 1.0 as possible [1]. In the case of the HOOF-Print assay, all isolates produced an amplified product at each of the eight loci. *Taq *DNA Polymerase adds a non-template nucleotide resulting in 2 products differing by 1-bp. With this protocol, the majority of amplicon products contain the extra nucleotide, but a significant portion of amplicons without the non-template nucleotide are also produced, resulting in the resolution of 2 peaks, 1-bp apart, by capillary electrophoresis. Nevertheless, since the repeat units increased in increments of 8-bp, allele assignment was clear-cut and an allele was assigned for all eight loci (see Additional file 1), giving the method a typeability index of 1.0. It should be noted that some strains were assigned an allele called M for Locus-6 and also for Locus-1. These isolates repeatedly produced single amplicons that were outside the predicted size range. For Locus-6, all M amplicons were the same size. Sequence analysis of the amplicons from several of these isolates showed a specific deletion in the flanking sequence region. All of the sequenced mutants produced the same aberrant sequence. Sequence analysis of the M allele at Locus-1 also showed a deletion in the flanking region of the DNA. Although these isolates did not fall in the normal expected range of alleles, they gave consistent results and could be assigned to a new allele that was called M for mutant.

### Reproducibility

Like typeability, it is critical that a typing method reliably produce the same result for a given sample. The ESGEM recommends a reproducibility index of R ≥ 0.95. This feature was continuously tested during the study since every isolate was independently tested twice. However, in a more structured evaluation, 20 isolates were analyzed in triplicate in a randomized sequence. In a blinded fashion, one individual was responsible for preparing the assay, performing the assay, and interpreting the results. The HOOF-Print Assay had an R = 0.998 at the locus level and an R = 0.983 at the composite fingerprint level. The reason that R was less than 1.0 was because in one test, Locus-6 was incorrectly recorded as containing Allele-2 instead of Allele-5. This was an obvious clerical error, not an experimental failure, since the raw data clearly showed Allele-5 as the only form present in the sample. Nevertheless, the assay easily met the suggested limit for reproducibility.

### Allelic diversity among the HOOF-Print loci

The *B*. *abortus *strains exhibited extensive variability in the number and range of alleles at each locus (Table 1). Similar levels of locus diversity were observed among each of the three biovars individually and within the test population as a whole. For example, Loci 5 and 8 had very little diversity, regardless of the biovar designation. By contrast, Locus-7 is highly diverse in all three biovars, with the same alleles occurring in multiple biovars.

Among the loci with multiple alleles, the distribution of alleles by size resembled an asymmetrical bell shaped curve skewed towards smaller repeat numbers (data not shown). This pattern of distribution is consistent with step-wise mutations resulting from SSM. The overall trend in allele frequency is toward short strings of repeats ranging from 2 to 5 repeat units per locus.

### Power of discrimination

A fundamental question addressed in epidemiological investigations is whether the outbreak strain is derived from or genetically related to the proposed index strain. The answer requires a method for differentiation of genetically related and unrelated strains. The more genetic markers that are available to define isolates, the easier it becomes to discriminate among related and unrelated strains. Therefore, the discriminatory power of a test indicates how successful the test will be in identifying genetic relationships among strains. The ESGEM recommends evaluating a large group (≥ 100 samples) of genetically diverse samples by the Hunter-Gaston Discrimination Index (HGDI) [21], and proposes as a limit that HGDI is ≥ 0.95. In other words, for any two randomly-chosen, unrelated isolates, there is a 95% or greater probability that they will be placed in separate groups. Since the HGDI is heavily influenced by the level of genetic diversity within the test population, our test population consisted of only one isolate per herd to avoid over-representation of related fingerprint profiles. To assess the demonstrable diversity within the less common biovars, a disproportionate number of randomly selected *B*. *abortus *biovar-2 and biovar-4 isolates was included. Despite all attempts to use genetically unrelated isolates, the level of genetic relatedness among outbreaks from the different herds is not known, and some isolates may be epidemiologically linked.

The discriminatory power of the conventional typing method for *Brucella*, biovar typing, was examined. The calculated HGDI for the total test population based solely on biovar typing was 0.60. It should be noted that this value is artificially high due to disproportionate representation of biovar-2 and biovar-4 isolates in the test population. Historically, about 85% of *B*. *abortus *infections in the U.S. were caused by biovar 1, which would result in an HGDI ≈ 0.2. Even with the disproportionate biovar representation, the discrimination index is well below the recommended power of discrimination, ≥ 0.95, but for many years it has been the only subtyping method available.

The discriminatory power of HOOF-Print genotyping was determined for each of the three biovar subpopulations and for the entire test population (n = 103). When the HGDI was calculated on a locus-by-locus basis within the biovar specific subgroups and within the total test population, the results were variable, ranging from 0 (no discrimination; Locus-8, all populations) to 0.93 (Locus-7; biovar-2 subgroup) as seen in Table 1. Although the locus-specific HGDI values varied slightly among the biovar subgroups, the trends were similar, indicating that HOOF-Print genotyping is equally discriminating for each biovar subgroup. On a single locus basis, none of the loci met the minimal discrimination level of ≥ 0.95.

When the alleles from all eight loci were combined into a composite HOOF-Print genotype, the test group of 103 samples exhibited 98 different fingerprint patterns (HGDI = 0.99). This is well above the recommended limit set by ESGEM. In only four cases did two isolates have the same fingerprint: isolates #16 and #17 [strains 1–2361 and 1–2385]; isolates #46 and #47 [strains 1–2421 and 1–2040]; isolates #79 and #96 [strains 98-0689 and 4-0213]; and isolates #102 and #103 [strains 6-0242 and 4-1303]; (see Additional file 1, highlighted in colored pairs). One field isolate, #25 [strain1-2052] matched the fingerprint pattern for the vaccine strain S19. Independent analysis of phenotypic and biochemical characteristics also identified this isolate as vaccine strain S19. Another field isolate, #31 [strain 1-2050], was previously characterized by biovar typing as vaccine strain S19. While the fingerprint pattern for this isolate was not an exact match for the fingerprint patterns for the other S19 samples, it differed by only 1 repeat unit at Locus-1. This type of difference is consistent with micro-evolution from a step-wise mutation event.

### Cluster analysis of HOOF-Print genotypes from the test populations

**Figure 1:**
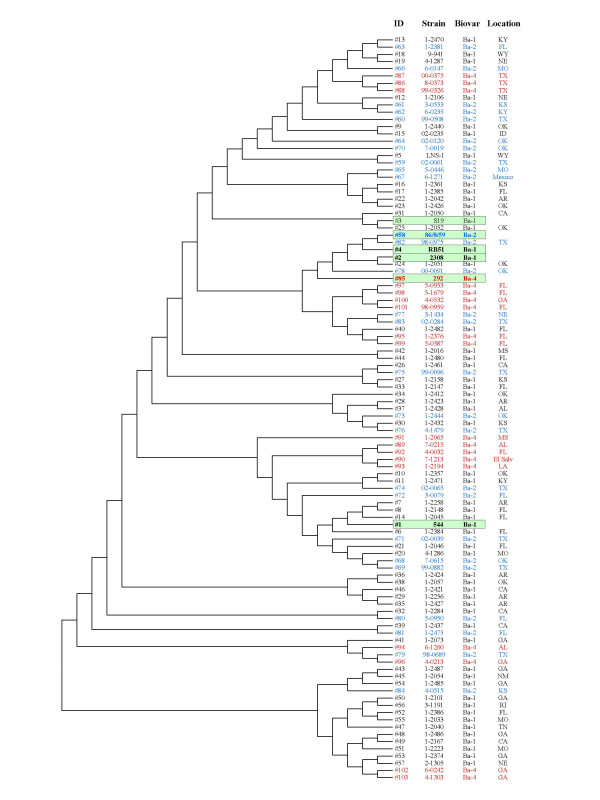
**Neighbor-joining dendrogram of clustered HOOF-Print genotypes. **Genotyped were clustered into groups based on the differences in the numbers of repeat units at the eight VNTR loci. Biovars are differentiated by colored text: black = biovar-1; green = biovar-2 and red = biovar-4.

The HOOF-Print assay was designed to complement epidemiological investigations. Genotypic similarities are assumed to demonstrate genetic linkage among related lineages, and conversely, related lineages would be expected to have genotypic similarities. However, since the mutation rates for the selected loci among *Brucella *strains have yet to be determined, the evolutionary distance defined by HOOF-Print genotyping is unclear. We used cluster analysis to see if the HOOF-Print genotypes could be used to infer long and short term evolutionary history. Pairwise genetic distances within the total 103 strain test population were calculated from the absolute difference in repeat units at each of the eight loci, consistent with the stepwise model of mutation. A dendrogram was created by the neighbor-joining method (Figure 1). The clusters appear to be independent of the time or location of strain isolation (e.g. isolates #18 and #19 that were isolated in 1999 and 1994, respectively). This is not surprising since the test population is presumably composed of unrelated strains. As expected, the few strains known to be genetically related did cluster appropriately (e.g. field isolates of the vaccine strain S19; RB51 and its parental strain 2308). Unfortunately, epidemiological information and comprehensive histories are not routinely submitted with diagnostic samples and so specific information about the selected test isolates was not available, making it impossible to assess the validity of the cluster results for these data. If VNTR polymorphism in *Brucella *is generated by a mechanism other than the stepwise mutation model, then the genetic relationships proposed in Figure 1 could be invalid.

### Distribution of HOOF-Print genotypes and alleles by geographic region

We wanted to see if there was a connection between geographic region and the HOOF-Print genotypes or alleles. The multilocus genotype clusters shown in the dendrogram in (Figure 1) do not correspond to specific states or regions. However, because multilocus genotyping is so highly discriminating, nearly every HOOF-Print genotype that was identified is unique. It is possible that the rapid evolution of the most variable loci could potentially mask regional influences in genotype composition. To detect regional relationships at the locus level, the alleles for each locus were examined for asymmetrical geographic distribution. To simplify the data and assure that sufficient data was available for statistical analysis, the US was divided into 2 regions: the east and the west, separated by the Mississippi River. For loci that contain a large number of alleles, the alleles were grouped so that no more that 4 or 5 groups were compared. Only data from *B. abortus *field strains from the US were included (n = 95). The data for each locus was cross tabulated and analyzed by the chi-square test statistic to see if a statistically significant association between alleles and geographic region could be demonstrated. The data are presented in Table 2 and Additional file 2. Note that for Locus-6, Fisher's exact test was used instead of the chi-square test, because of the small number of strains carrying alleles with more than 2 repeat units. The data indicate that within most loci there are no significant associations between alleles and geographic region (*p *values ranging from 0.1739 to 0.8563). For Locus 1, there may be a slight imbalance in distribution, with smaller alleles (7 or less repeat units) appearing more often in the west and larger alleles (8 or more repeat units) appearing more often in the east (*p *= 0.0400).

While it is reasonable to expect a relationship between genotypes/alleles and geographic region, several factors can affect geographic distributions. In the case of brucellosis, the government sponsored eradication program is drawing to a close. As a result, the incidence of *Brucella *has decreased dramatically to nearly complete eradication. Many states have been disease free for decades. Therefore, the initial source of a new infection is often found outside of the local area and involves the importation of diseased cattle from elsewhere. In a few areas, transmission from wildlife to cattle has become the leading source of disease outbreaks. Only a very small number of residual endemic bovine infections continue to be identified and eradicated. As the number of infected herds decrease, each outbreak is more isolated and can be quickly contained so that subsequent regional spread of the associated genotype is less likely. Therefore, it is likely that with so the few infected herds, the remaining *B*. *abortus *genotypes in the US are no longer representative of the natural distribution of genotypes that were present prior to the eradication program.

Finally, the selection criteria for the test population used in this study were designed to maximize the number of unrelated strains. Strains were selected from a large number of different states, and when possible, only one isolate from each city was included. Thus, possible links between alleles and geographic region may not be represented in the test population.

### Comparison of biovar typing and HOOF-Print genotyping

The fourth performance criterion recommended by the ESGEM is concordance of the typing results with the results of other typing methods. When HOOF-Print genotyping was compared with conventional biovar typing, the most obvious difference was the high discriminatory power of the genotyping method and low discriminatory power of the biovar typing method. This complicates the direct comparison of typing results because the numbers of subtypes generated vary greatly between the two methods. In the dendrogram shown in Figure 1, all 103 strains were clustered by genotypic similarity. However, the hierarchical groups assembled by genotype were not homogeneous for biovar type. Most genotype clusters contain an assortment of biovars (shown by color coding in Figure 1), although some small clusters of biovars were found (e.g. biovar-4 isolates #89, 90, 91, 92 and 93). It is important to keep in mind that the dendrogram in Figure 1 is only one of many possible arrangements of the data. Furthermore, the method used for clustering the data was based on the step-wise model of mutation. If a different mechanism or a combination of mechanisms is involved, then the clustering parameters used in this study do not apply and the clusters are invalid.

In the most extreme case, two isolates with identical fingerprints were identified as belonging to different biovars (see Additional file 1, isolate #79 – biovar 2 and isolate #96 – biovar 4, highlighted in yellow). This result was somewhat surprising, considering the clonal nature of *Brucella*, but there are at least two possible explanations for this observation. One possible cause is homoplasy, resulting from convergent evolution among unrelated strains. The hypermutability of VNTR loci results in continuous micro-evolution, and HOOF-Print genotyping reveals the most recent mutation events in a strain's genetic history. Even among the most variable loci in this study, a limited repertoire of alleles was found. Although the total number of allelic combinations is large, random convergence of genotype patterns between genetically unrelated strains is likely to occur occasionally. Incorporation of additional polymorphic loci into the assay may help resolve inconsistencies caused by homoplasy.

Alternatively, the strain could have typed as a different biovar type due to mutation(s) in genes that affect biovar phenotype. Only two differences distinguish *B*. *abortus *biovar 2 and biovar 4: biovar 2 strains are A-antigen dominant/fuchsin dye sensitive while biovar 4 strains are M-antigen dominant/fuchsin dye insensitive [16]. Both traits are associated with the bacterial surface and may require common genes. Although spontaneous mutations are much less common than VNTR polymorphisms, atypical biovar profiles are occasionally found among field isolates. Thus, this argument cannot be ruled out without further study.

## Conclusion

The HOOF-Print Assay is a rapid, easy to perform technique for subtyping *B. abortus *strains. When the assay was evaluated with a test population consisting of a large number of unrelated, naturally occurring field isolates, the selected tandem repeat loci displayed considerable polymorphism as evidenced by the large number of alleles found at many of the loci. The study demonstrates that the HOOF-Print assay meets or exceeds the minimum limits recommended by the ESGEM for epidemiological typing, based on the performance criteria: typeability, reproducibility, and power of discrimination. When compared to conventional biovar typing, HOOF-Print genotyping is considerably more discriminating. However, some inconsistencies with conventional biovar typing were observed. Caution will be needed in interpreting the data, to prevent drawing incorrect conclusions from artificial genotypic similarity caused by homoplasy. Incorporation of additional polymorphic loci may help identify convergent evolution among unrelated strains. In practice, HOOF-Print genotyping should be utilized as a complement to conventional epidemiological investigations into the short-term history of localized brucellosis outbreaks. In the future, we plan to look at genotypic variability among related isolates to better understand how HOOF-Print genotypes evolve and to experimentally measure the VNTR mutation rates among the polymorphic *B. abortus *loci.

## Methods

Disclaimer: Mention of trade names or commercial products in this article is solely for the purpose of providing specific information and does not imply recommendation or endorsement by the U.S. Department of Agriculture.

### Isolation and characterization of the bacterial strains used in this study

The bacterial field strains used in this study (see Additional file 1) were originally isolated in the Bacterial Diagnostic Laboratory at the USDA National Veterinary Services Laboratories, Ames, IA, as part of the USDA Brucellosis Eradication Program. The suspect bacteria were subcultured, identified by conventional microbiological tests and biotyped by additional biochemical and phenotypic characteristics [16] prior to archiving at -70°C. The type strains were originally obtained from the American Type Culture Collection bank and stored at -70°C until propagated.

The selected strains were thawed, grown, harvested and preserved in 66% methanol. Many of the isolates were retrieved and prepared specifically for this study while other strains had been preserved in methanol for a number of years with no apparent DNA degradation.

### HOOF-Print analysis

The HOOF-Print technique was performed as previously described [17] with minor modifications as follows: PCR amplification was performed with 1 unit of FastStart Taq polymerase (cat no. 2-032-902; Roche Biochemicals, Indianapolis, IN) in 50-mM Tris, 10-mM KCl, 1.5-mM MgCl_2_, and 5-mM (NH_4_)_2_SO_4_, pH 8.3, with the addition of dGTP, dATP, dCTP and dTTP at 250-μM each, primers at 250-nM each, and GC-Rich Resolution Solution (included with the polymerase) at 1X concentration, per manufacturer's instructions. Primer pairs [17] consisted of one primer fluorescently labeled with HEX, FAM or NED and one unlabeled primer. Both primers were designed to anneal to the conserved sequences flanking the repeat region. Amplification was done one locus at a time (8 assays per sample) or multiplexed with up to three primer sets per reaction (with a different dye for each primer set). Cycling parameters were as follows: 95°C for 5 min to activate the modified polymerase, followed by 37 cycles of 95°C for 30 sec, 53°C for 30 sec and 75°C for 45 sec; and ending with a 60-min incubation at 75°C to promote maximal addition of the non-templated nucleotide to the amplicons. The higher extension temperature increased amplicon yield, possibly by denaturing short hairpin formations in the target sequence. The amplified samples were maintained at 4°C prior to analysis.

The degree of amplification success was monitored by gel electrophoresis through 4% agarose. Before size analysis, the fluorescent amplicons were diluted in water, usually in a 1:100 or 1:200 ratio, then separated by capillary electrophoresis on an ABI Prism 3100 Genetic Analyzer with GeneScan-500 [ROX] size markers (cat. no. 401734; Applied BioSystems, Foster City, CA). Data were collected and the amplicon sizes were determined with GeneScan Data Analysis Software, ver 3.7 (Applied BioSystems). The number of repeat copies was deduced from the amplicon size.

For some isolates, more than one product was reproducibly synthesized for a given locus, typically differing by a single repeat unit. This phenomenon, which was especially common in reference strains, is thought to be the result of micro-evolution. Minor heterogeneity at some loci was not unexpected since many of these samples were primary isolates that had not been propagated clonally. In these cases the dominant allele was used for analysis.

### Reproducibility analysis

A reproducibility evaluation was integrated into the protocol by analyzing every sample twice in independent assays. To formally evaluate the reproducibility of the HOOF-Print technique an additional blinded survey was performed. Twenty isolates were prepared in triplicate and randomized in order. A different individual prepared the reaction mixes, performed the assay and analyzed the data, unaware of the identities of the samples.

### Statistical analyses

The following statistical analyses were performed as suggested by the ESGEM [1].

*Typeability *is the success in determining an unambiguous type for each isolate, calculated by the formula  where *T *is the typeability, *N*_*t *_is the number of strains characterized by a complete array of alleles, and *N *is the size of the test population. If a complete 8-allele fingerprint can be determined for all strains in the test population, then *T *= 1.

*Reproducibility *is the ability to assign the same type to an isolate in independently repeated tests. It is calculated from the equation  where *R *is the reproducibility index, *N*_*r *_is the number of strains that are repeatedly assigned a single type and *N *is the total number in the test sample.

*Allele frequency *is the relative proportion of each allele, given as a percentage by the formula  where *n*_*j *_is the number of strains with the allele "*j*"; and *N *is the size of the test population.

The *Hunter-Gaston Discrimination Index *[21] measures the power of discrimination for a typing method by calculating the probability that two unrelated strains will be correctly assigned to different types. The equation used is

 where *DI *is the index of discrimination; *N *is the size of the

test population; *s *is the total number of alleles per locus or fingerprint patterns in the population, and *n*_*j *_is the number of isolates with the allele "*j*".

*Cluster and molecular evolutionary analyses *were conducted using MEGA version 2.1 [22]. A distance matrix was created using pairwise comparison of HOOF-Print genotypes from all of the 103 isolates in the test population. The genetic distance was calculated from the absolute difference in repeat units at each locus, assuming that each incremental change in repeat number represents an equal and independent mutation event. The distance matrix was used to make an unrooted tree using the neighbor-joining method [23].

*Statistical analysis of the distribution of alleles by geographic region *was done by creating a cross tabulation of each locus into two regions, defined as east or west of the Mississippi River. For the loci that contain a large number of alleles, the alleles were grouped so that no more than four or five groups were compared to make sure that enough data for each locus would be compared (see Additional file 2). Differences among the data were analyzed using the chi-square test statistic or, in the case of Locus-6, the Fisher's exact test (Table 2). Statistical analyses were performed with the SAS system version 8 (SAS Institute, Cary, N.C.) using the FREQ Procedure based on a sample size of 95. A *P *value < 0.05 was considered to be significant.

## List of abbreviations

PCR – polymerase chain reaction; bp – base pair; SSM – slip-strand mispairing; DI – discrimination index; HGDI – Hunter Gaston discrimination index; ESGEM – European Study Group on Epidemiological Markers; VNTR – variable number tandem repeats; MLVA – multilocus VNTR analysis; SSR – short sequence repeats.

## Authors' contributions

DRE isolated, cultivated, harvested and processed most of the bacterial strains used in this study; typed each strain by conventional biotyping methods and compiled the available background information for each isolate. BJB performed the HOOF-Print assay on all strains, determined the genotypes, performed most of the statistical analyses of the data, and wrote the manuscript. Both authors contributed to discussions about the data, read and approved the final manuscript.

## Supplementary Material

Additional File 1 ***Brucella *strains and HOOF-Print genotypes. **List of all of the bacterial isolates used in this study, including the biovar designation of the isolate, the location of the infected herd, the year of isolation, and the HOOF-Print genotype. Click here for file

Additional File 2 **Cross-tabulation of allelic distribution within the variable HOOF-Print loci by geographic region. **Statistical data from each of the six most variable loci are presented in cross-tabulations displaying the distribution of alleles between the eastern and western regions of the US. Click here for file

## Figures and Tables

**Table 1:** Allelic diversity among B. abortus VNTR loci

	**No. of Alleles**	**Range of Repeats**	**Discrimination Index^*a*^**
**Biovar 1 strains**			
Locus-1	14	2 – 14, M^*c*^	0.90
Locus-2	4	3 – 6	0.66
Locus-3	9	3 – 16	0.78
Locus-4	7	2 – 10	0.77
Locus-5	2	2 – 3	0.07
Locus-6	3	2 – 3, M	0.17
Locus-7	12	3 – 14	0.87
Locus-8	1	2	0.00
**Biovar 2 strains**			
Locus-1	8	2 – 10	0.80
Locus-2	5	3 – 7	0.71
Locus-3	8	2 – 13	0.86
Locus-4	6	2 – 7	0.78
Locus-5	2	2 – 5	0.14
Locus-6	4	2 – 4, M	0.39
Locus-7	12	3 – 15	0.93
Locus-8	1	2	0.00
**Biovar 4 strains**			
Locus-1	10	2 – 12	0.92
Locus-2	2	4 – 5	0.53
Locus-3	5	1 – 6	0.74
Locus-4	5	2 – 6	0.77
Locus-5	1	2	0.00
Locus-6	2	2, M	0.43
Locus-7	9	3 – 12	0.90
Locus-8	1	2	0.00
**All strains^*b*^**			
Locus-1	14	2 – 14, M	0.87
Locus-2	5	3 – 7	0.67
Locus-3	11	1 – 16	0.82
Locus-4	7	2 – 13	0.78
Locus-5	3	2 – 5	0.04
Locus-6	5	2 – 5, M	0.40
Locus-7	13	3 – 15	0.89
Locus-8	1	2	0.00

*a*-The Discrimination Index was calculated for each locus by the method of Hunter and Gaston as described in the Materials and methods section; *b *-These numbers were calculated from the entire 103-sample test population. *c *– "M" indicates an atypical (mutant) allele.

**Table 2:** Statistical analysis of the allelic distributions within each VNTR locus by geographic region^*a*^

**VNTR Locus**	***P *value**
**Locus-1**	0.0400
**Locus-2**	0.1739
**Locus-3**	0.8365
**Locus-4**	0.4147
**Locus-6**	0.2824
**Locus-7**	0.8563

*a *– See Additional file 2 for the data that was used in the analysis.
