# Supplementary material for: Evaluation of the HOOF-Print assay for typing Brucella abortus strains isolated from cattle in the United States: results with four performance criteria
Source: BMC Microbiol. 2005 Jun 23;5:37. doi: 10.1186/1471-2180-5-37 (PMC1183211; doi:10.1186/1471-2180-5-37)
Supplement: Additional File 1 — Brucella strains and HOOF-Print genotypes. List of all of the bacterial isolates used in this study, including the biovar designation of the isolate, the location of the infected herd, the year of isolation, and the HOOF-Print genotype. [file 1471-2180-5-37-S1.doc]

| **ID #** | **NADC #** | **NVSL #** | **Biovar** | **State** | **City** | **Herd*^a^*** | **Year Isolated** | **Locus 1*^b^*** | **Locus 2** | **Locus 3** | **Locus 4** | **Locus 5** | **Locus 6** | **Locus 7** | **Locus 8** |
| --- | --- | --- | --- | --- | --- | --- | --- | --- | --- | --- | --- | --- | --- | --- | --- |
| 1 *^cd^* | 544 | NA*^e^* | Bv-1 | ref *^f^* | type strain |  |  | 3 | 4 | 6 | 5 | 2 | 2 | 4 | 2 |
| 2 *^c^* | 2308 | NA | Bv-1 | ref | challenge strain |  |  | 4 | 4 | 6 | 2 | 2 | 2 | 10 | 2 |
| 3 *^c^* | S19 | NA | Bv-1 | ref | vaccine strain |  |  | 5 | 4 | 4 | 2 | 2 | 2 | 8 | 2 |
| 4 *^c^* | RB51 | NA | Bv-1 | ref | vaccine strain |  |  | 5 | 4 | 4 | 2 | 2 | 2 | 11 | 2 |
| 5 | NA | LNS-1 | Bv-1 | WY | Muddy Creek | A | 2004 | M *^g^* | 4 | 3 | 3 | 2 | 2 | 7 | 2 |
| 6 | 1065 | 1-2384 | Bv-1 | FL |  | A | 1991 | 2 | 3 | 4 | 5 | 2 | 2 | 4 | 2 |
| 7 | 1044 | 1-2258 | Bv-1 | AR |  | A | 1991 | 2 | 3 | 6 | 3 | 2 | 2 | 3 | 2 |
| 8 *^cd^* | 1047 | 1-2148 | Bv-1 | FL | Leesburg | B | 1991 | 2 | 3 | 7 | 3 | 2 | 2 | 5 | 2 |
| 9 | 1087 | 1-2440 | Bv-1 | OK |  | A | 1991 | 2 | 4 | 3 | 5 | 2 | 3 | 10 | 2 |
| 10 | 1070 | 1-2357 | Bv-1 | OK | Haskell | B | 1991 | 2 | 4 | 3 | 6 | 2 | 2 | 6 | 2 |
| 11 | 1090 | 1-2471 | Bv-1 | KY | McKee | A | 1991 | 2 | 4 | 4 | 6 | 2 | 3 | 7 | 2 |
| 12 | 1035 | 1-2106 | Bv-1 | NE | Palmer | A | 1991 | 2 | 4 | 5 | 4 | 2 | 2 | 10 | 2 |
| 13 | 1089 | 1-2470 | Bv-1 | KY |  | B | 1991 | 2 | 4 | 5 | 4 | 2 | 3 | 12 | 2 |
| 14 *^c^* | 1007 | 1-2045 | Bv-1 | FL |  | C | 1991 | 2 | 4 | 9 | 4 | 2 | 2 | 5 | 2 |
| 15 | NA | 02-0235 | Bv-1 | ID |  | A | 2002 | 2 | 5 | 3 | 10 | 2 | 2 | 10 | 2 |
| 16 | 1052 | 1-2361 | Bv-1 | KS | Colwich | A | 1991 | 2 | 5 | 4 | 2 | 2 | 2 | 8 | 2 |
| 17 *^c^* | 1066 | 1-2385 | Bv-1 | FL | Webster | D | 1991 | 2 | 5 | 4 | 2 | 2 | 2 | 8 | 2 |
| 18 | NA | 9-941 | Bv-1 | WY |  | B | 1999 | 2 | 5 | 4 | 4 | 2 | 2 | 14 | 2 |
| 19 | 1290 | 4-1287 | Bv-1 | NE | Boelus | B | 1994 | 2 | 4 | 6 | 3 | 2 | 2 | 13 | 2 |
| 20 | 1289 | 4-1286 | Bv-1 | MO | Passaic | A | 1994 | 2 | 6 | 3 | 6 | 2 | 3 | 3 | 2 |
| 21 *^cd^* | 1008 | 1-2046 | Bv-1 | FL | Marathon | E | 1991 | 3 | 5 | 3 | 4 | 2 | 2 | 3 | 2 |
| 22 | 1006 | 1-2042 | Bv-1 | FL | Brooksville | F | 1991 | 3 | 5 | 5 | 2 | 2 | 2 | 9 | 2 |
| 23 | 1079 | 1-2426 | Bv-1 | AR | Little Rock (Lab) | B | 1991 | 3 | 6 | 5 | 2 | 2 | 2 | 8 | 2 |
| 24 | 1011 | 1-2051 | Bv-1 | OK |  | C | 1991 | 4 | 4 | 7 | 2 | 2 | 2 | 11 | 2 |
| 25 | 1012 | 1-2052 | Bv-1 | OK |  | D | 1991 | 5 | 4 | 4 | 2 | 2 | 2 | 8 | 2 |
| 26 | 1094 | 1-2461 | Bv-1 | CA | Chino | A | 1991 | 5 | 5 | 3 | 4 | 2 | 2 | 8 | 2 |
| 27 | 1048 | 1-2158 | Bv-1 | KS | Mulvane | B | 1991 | 5 | 5 | 3 | 6 | 2 | 2 | 7 | 2 |
| 28 *^cd^* | 1076 | 1-2423 | Bv-1 | AR | Little Rock (Lab) | C | 1991 | 5 | 5 | 6 | 4 | 2 | 2 | 8 | 2 |
| 29 | 1042 | 1-2256 | Bv-1 | AR | Mansfield | D | 1991 | 5 | 6 | 4 | 6 | 2 | 2 | 3 | 2 |
| 30 *^cd^* | 1085 | 1-2432 | Bv-1 | KS | Wier | C | 1991 | 5 | 6 | 8 | 2 | 2 | 2 | 8 | 2 |
| 31 | 1010 | 1-2050 | Bv-1 | CA | Ontario | B | 1991 | 6 | 4 | 4 | 2 | 2 | 2 | 8 | 2 |
| 32 | 1068 | 1-2284 | Bv-1 | CA | Corona | C | 1991 | 6 | 5 | 3 | 4 | 2 | 2 | 5 | 2 |
| 33 *^cd^* | 1046 | 1-2147 | Bv-1 | FL | Okeechobee | G | 1991 | 6 | 5 | 3 | 6 | 2 | 2 | 7 | 2 |
| 34 | 1093 | 1-2412 | Bv-1 | OK | Poteau | E | 1991 | 6 | 5 | 8 | 7 | 2 | 2 | 6 | 2 |
| 35 | 1080 | 1-2427 | Bv-1 | AR | Little Rock (Lab) | E | 1991 | 6 | 6 | 4 | 6 | 2 | 2 | 3 | 2 |
| 36 | 1077 | 1-2424 | Bv-1 | AR | Little Rock (Lab) | F | 1991 | 7 | 5 | 5 | 6 | 2 | 2 | 5 | 2 |
| 37 *^cd^* | 1081 | 1-2428 | Bv-1 | AL | Huntsville | A | 1991 | 7 | 5 | 6 | 4 | 2 | 2 | 8 | 2 |
| 38 | 1028 | 1-2057 | Bv-1 | OK | Meeker | F | 1991 | 7 | 6 | 5 | 5 | 2 | 2 | 6 | 2 |
| 39 | 1086 | 1-2437 | Bv-1 | CA | Lakeview | D | 1991 | 8 | 4 | 3 | 4 | 2 | 2 | 5 | 2 |
| 40 | 1102 | 1-2482 | Bv-1 | FL | W. Palm Beach | H | 1991 | 8 | 5 | 4 | 4 | 2 | 2 | 8 | 2 |
| 41 | 1022 | 1-2073 | Bv-1 | GA | Kingston | A | 1991 | 8 | 5 | 13 | 2 | 2 | 2 | 3 | 2 |
| 42 | 1001 | 1-2016 | Bv-1 | MS | Carrolton | A | 1991 | 9 | 4 | 3 | 6 | 2 | 2 | 8 | 2 |
| 43 | 1106 | 1-2487 | Bv-1 | GA | Kingston | B | 1991 | 9 | 5 | 16 | 2 | 2 | 2 | 3 | 2 |
| 44 | 1101 | 1-2480 | Bv-1 | FL | Okeechobee | I | 1991 | 9 | 6 | 4 | 6 | 2 | 2 | 4 | 2 |
| 45 | 1021 | 1-2054 | Bv-1 | NM |  | A | 1991 | 10 | 4 | 5 | 2 | 3 | 2 | 7 | 2 |
| 46 *^cd^* | 1074 | 1-2421 | Bv-1 | CA | San Jacinto | E | 1991 | 10 | 5 | 3 | 4 | 2 | 2 | 6 | 2 |
| 47 | 1020 | 1-2040 | Bv-1 | TN | Lebanon | A | 1991 | 10 | 5 | 3 | 4 | 2 | 2 | 6 | 2 |
| 48 | 1105 | 1-2486 | Bv-1 | GA |  | A | 1991 | 11 | 4 | 3 | 2 | 2 | 2 | 8 | 2 |
| 49*^d^* | 1032 | 1-2167 | Bv-1 | CA | Chino | F | 1991 | 11 | 5 | 3 | 4 | 2 | 2 | 5 | 2 |
| 50 | 1027 | 1-2101 | Bv-1 | GA | Sylvester | B | 1991 | 12 | 4 | 4 | 2 | 2 | 2 | 6 | 2 |
| 51 | 1038 | 1-2223 | Bv-1 | MO | Morgan | B | 1991 | 12 | 5 | 3 | 6 | 2 | M | 8 | 2 |
| 52 | 1067 | 1-2386 | Bv-1 | FL | Alapala | J | 1991 | 12 | 6 | 6 | 2 | 2 | 2 | 3 | 2 |
| 53 | 1057 | 1-2374 | Bv-1 | GA | Ashburn | C | 1991 | 13 | 4 | 4 | 2 | 2 | 2 | 5 | 2 |
| 54 | 1104 | 1-2485 | Bv-1 | GA |  | D | 1991 | 14 | 4 | 3 | 2 | 2 | 2 | 8 | 2 |
| 55 | 1005 | 1-2033 | Bv-1 | MO | Versailles | C | 1991 | 14 | 5 | 3 | 6 | 2 | 2 | 8 | 2 |
| 56 | 1184 | 3-1191 | Bv-1 | RI |  | A | 1993 | 8 | 5 | 3 | 4 | 2 | 2 | 12 | 2 |
| 57 | 1185 | 2-1305 | Bv-1 | NE |  | C | 1992 | 8 | 5 | 4 | 2 | 2 | 2 | 5 | 2 |
| 58*^cd^* | 86/8/59 | NA | Bv-2 | ref | type strain |  |  | 7 | 3 | 2 | 2 | 2 | M | 3 | 2 |
| 59 | NA | 02-0061 | Bv-2 | TX |  | A | 2002 | 2 | 3 | 3 | 4 | 2 | 2 | 7 | 2 |
| 60 | NA | 99-0508 | Bv-2 | TX | Palo Pinto | B | 1999 | 2 | 3 | 4 | 4 | 2 | 2 | 9 | 2 |
| 61 | NA | 3-0553 | Bv-2 | KS | Penacosa | D | 1993 | 2 | 3 | 5 | 3 | 2 | 2 | 10 | 2 |
| 62 | NA | 6-0235 | Bv-2 | KY | Benton | C | 1996 | 2 | 3 | 5 | 4 | 2 | 3 | 9 | 2 |
| 63*^d^* | 1062 | 1-2381 | Bv-2 | FL | Canal Pt | K | 1991 | 2 | 3 | 5 | 5 | 2 | 4 | 12 | 2 |
| 64 | NA | 02-0120 | Bv-2 | OK | Boswell | G | 2002 | 2 | 4 | 2 | 5 | 2 | 2 | 8 | 2 |
| 65 | NA | 5-0446 | Bv-2 | MO | Rich Hill | D | 1995 | 2 | 4 | 4 | 3 | 2 | 2 | 8 | 2 |
| 66 | NA | 6-0147 | Bv-2 | MO | Jefferson City - Lab | E | 1996 | 2 | 4 | 5 | 4 | 2 | 2 | 11 | 2 |
| 67 | NA | 6-1271 | Bv-2 | MEX |  |  | 1996 | 2 | 4 | 6 | 3 | 2 | 2 | 8 | 2 |
| 68 | NA | 7-0615 | Bv-2 | OK | Oklahoma City - Lab | H | 1997 | 2 | 5 | 2 | 4 | 2 | 3 | 3 | 2 |
| 69 | NA | 99-0882 | Bv-2 | TX |  | C | 1999 | 2 | 6 | 2 | 4 | 2 | 3 | 3 | 2 |
| 70 | NA | 7-0019 | Bv-2 | OK | Council Mill | I | 1997 | 3 | 4 | 3 | 4 | 2 | 2 | 9 | 2 |
| 71 | NA | 02-0039 | Bv-2 | TX |  | D | 2002 | 3 | 4 | 3 | 7 | 2 | 2 | 4 | 2 |
| 72 | NA | 3-0079 | Bv-2 | FL | Clewiston | L | 1993 | 3 | 5 | 4 | 5 | 2 | 2 | 5 | 2 |
| 73*^d^* | 1088 | 1-2444 | Bv-2 | OK | Waurika | J | 1991 | 3 | 5 | 13 | 4 | 2 | 2 | 10 | 2 |
| 74 | NA | 02-0065 | Bv-2 | TX |  | E | 2002 | 4 | 4 | 3 | 6 | 2 | 2 | 6 | 2 |
| 75 | NA | 99-0096 | Bv-2 | TX | Gilman | F | 1999 | 5 | 6 | 2 | 5 | 2 | 2 | 8 | 2 |
| 76 | NA | 4-1479 | Bv-2 | TX | Eagle Pass | G | 1994 | 5 | 7 | 8 | 5 | 5 | M | 9 | 2 |
| 77 | NA | 3-1434 | Bv-2 | NE |  | D | 1993 | 6 | 4 | 2 | 2 | 2 | 2 | 15 | 2 |
| 78 | NA | 00-0091 | Bv-2 | OK | Wilson | K | 2000 | 6 | 4 | 6 | 4 | 2 | 2 | 10 | 2 |
| 79 | NA | 98-0689 | Bv-2 | TX | Groesbeck | H | 1998 | 6 | 5 | 5 | 2 | 2 | 2 | 5 | 2 |
| 80 | NA | 5-0950 | Bv-2 | FL | Lake Wales | M | 1995 | 6 | 7 | 3 | 4 | 2 | 2 | 4 | 2 |
| 81*^d^* | 1096 | 1-2475 | Bv-2 | FL | Wauchula | N | 1991 | 7 | 3 | 3 | 3 | 2 | 2 | 6 | 2 |
| 82 | NA | 98-0975 | Bv-2 | TX |  | I | 1998 | 7 | 4 | 4 | 2 | 2 | 2 | 10 | 2 |
| 83 | NA | 02-0284 | Bv-2 | TX |  | J | 2002 | 9 | 4 | 4 | 2 | 2 | 2 | 13 | 2 |
| 84 | NA | 4-0515 | Bv-2 | KS | Parsons | E | 1994 | 10 | 4 | 7 | 5 | 2 | M | 5 | 2 |
| 85*^cd^* | 292 | NA | Bv-4 | ref | type strain |  |  | 7 | 4 | 1 | 2 | 2 | 5 | 3 | 2 |
| 86 | NA | 98-0373 | Bv-4 | TX | Synder | K | 1998 | 2 | 4 | 4 | 5 | 2 | M | 12 | 2 |
| 87 | NA | 00-0375 | Bv-4 | TX |  | L | 2000 | 2 | 4 | 4 | 6 | 2 | 2 | 11 | 2 |
| 88 | NA | 99-0326 | Bv-4 | TX |  | M | 1999 | 2 | 4 | 4 | 6 | 2 | M | 11 | 2 |
| 89 | NA | 7-0215 | Bv-4 | AL | Bayou Labatre | B | 1997 | 3 | 5 | 1 | 5 | 2 | M | 5 | 2 |
| 90 | NA | 7-1213 | Bv-4 | El Salv. |  |  | 1997 | 4 | 4 | 5 | 6 | 2 | M | 4 | 2 |
| 91 | 1014 | 1-2065 | Bv-4 | MS | Winona | B | 1991 | 5 | 4 | 4 | 5 | 2 | 2 | 5 | 2 |
| 92 | NA | 4-0032 | Bv-4 | FL | Bradenton | O | 1994 | 5 | 5 | 2 | 4 | 2 | M | 6 | 2 |
| 93 | 1037 | 1-2194 | Bv-4 | LA | Oak Grove | A | 1991 | 5 | 5 | 4 | 4 | 2 | M | 3 | 2 |
| 94 | NA | 6-1260 | Bv-4 | AL | Greensboro | C | 1996 | 5 | 5 | 6 | 2 | 2 | 2 | 5 | 2 |
| 95 | 1058 | 1-2376 | Bv-4 | FL | Zolfo Springs | P | 1991 | 6 | 4 | 2 | 4 | 2 | M | 12 | 2 |
| 96 | NA | 4-0213 | Bv-4 | GA | Sylvester | E | 1994 | 6 | 5 | 5 | 2 | 2 | 2 | 5 | 2 |
| 97 | NA | 5-1679 | Bv-4 | FL | Lakeland | Q | 1995 | 8 | 5 | 4 | 4 | 2 | M | 9 | 2 |
| 98 | NA | 5-0953 | Bv-4 | FL | Dade City | R | 1995 | 8 | 5 | 4 | 4 | 2 | M | 10 | 2 |
| 99 | NA | 5-0387 | Bv-4 | FL | Arcadia | S | 1995 | 9 | 4 | 2 | 5 | 2 | M | 12 | 2 |
| 100 | NA | 4-0532 | Bv-4 | GA | Warwick | F | 1994 | 9 | 5 | 5 | 4 | 2 | M | 9 | 2 |
| 101 | NA | 98-0959 | Bv-4 | FL | Green Cove Spring | T | 1998 | 10 | 4 | 5 | 3 | 2 | M | 9 | 2 |
| 102 | NA | 6-0242 | Bv-4 | GA |  | G | 1996 | 12 | 5 | 5 | 4 | 2 | M | 8 | 2 |
| 103 | NA | 4-1303 | Bv-4 | GA | Warwick | H | 1994 | 12 | 5 | 5 | 4 | 2 | M | 8 | 2 |

*a* - Herd is an arbitrary designation to differentiate among multiple herds from the same state; *b* - Locus columns identify the allele (number of complete tandem repeat units) determined for each locus within the designated strain; *c* - HOOF-Print for this strain was previously published [17]; *d* - HOOF-Print for this strain was previously published [20]; *e* - “NA” indicates that the strain is not a member of the specified culture collection; *f* - “ref” identifies common laboratory reference or vaccine strains; *g* - “M” indicates an atypical (mutant) allele. Under cities, “lab” indicates that the isolate was submitted from a regional diagnostic laboratory without additional information regarding its specific history. Green background indicates a common laboratory strain. Strains with identical fingerprints are indicated by matching colored backgrounds in the alleles’ columns.
