# Supplementary material for: Evaluation of the HOOF-Print assay for typing Brucella abortus strains isolated from cattle in the United States: results with four performance criteria
Source: BMC Microbiol. 2005 Jun 23;5:37. doi: 10.1186/1471-2180-5-37 (PMC1183211; doi:10.1186/1471-2180-5-37)
Supplement: Additional File 2 — Cross-tabulation of allelic distribution within the variable HOOF-Print loci by geographic region. Statistical data from each of the six most variable loci are presented in cross-tabulations displaying the distribution of alleles between the eastern and western regions of the US. [file 1471-2180-5-37-S2.doc]

| **Cross-tabulation of Locus-1 by region** | | | | | |
| --- | --- | --- | --- | --- | --- |
| **Frequency Percent Row Pct Col Pct** | **Alleles 2, 3 and 4** | **Alleles 5, 6 and 7** | **Alleles 8, 9 and 10** | **Alleles >10**  **Allele “M”** | **Total** |
| **West** | 25 26.32 45.45 67.57 | 19 20.00 34.55 67.86 | 6 6.32 10.91 33.33 | 5 5.26 9.09 41.67 | 55 57.89 |
| **East** | 12 12.63 30.00 32.43 | 9 9.47 22.50 32.14 | 12 12.63 30.00 66.67 | 7 7.37 17.50 58.33 | 40 42.11 |
| **Total** | 37 38.95 | 28 29.47 | 18 18.95 | 12 12.63 | 95 100.00 |

| **Cross-tabulation of Locus-2 by region** | | | | | |
| --- | --- | --- | --- | --- | --- |
| **Frequency Percent Row Pct Col Pct** | **Allele 3** | **Allele 4** | **Allele 5** | **Alleles 6 and 7** | **Total** |
| **West** | 4 4.21 7.27 44.44 | 24 25.26 43.64 66.67 | 18 18.95 32.73 47.37 | 9 9.47 16.36 75.00 | 55 57.89 |
| **East** | 5 5.26 12.50 55.56 | 12 12.63 30.00 33.33 | 20 21.05 50.00 52.63 | 3 3.16 7.50 25.00 | 40 42.11 |
| **Total** | 9 9.47 | 36 37.89 | 38 40.00 | 12 12.63 | 95 100.00 |

| **Cross-tabulation of Locus-3 by region** | | | | | | |
| --- | --- | --- | --- | --- | --- | --- |
| **Frequency Percent Row Pct Col Pct** | **Alleles 1 and 2** | **Allele 3** | **Allele 4** | **Allele 5** | **Alleles >5** | **Total** |
| **West** | 5 5.26 9.09 55.56 | 17 17.89 30.91 65.38 | 15 15.79 27.27 57.69 | 8 8.42 14.55 47.06 | 10 10.53 18.18 58.82 | 55 57.89 |
| **East** | 4 4.21 10.00 44.44 | 9 9.47 22.50 34.62 | 11 11.58 27.50 42.31 | 9 9.47 22.50 52.94 | 7 7.37 17.50 41.18 | 40 42.11 |
| **Total** | 9 9.47 | 26 27.37 | 26 27.37 | 17 17.89 | 17 17.89 | 95 100.00 |

| **Cross-tabulation of Locus 4 by region** | | | | | | |
| --- | --- | --- | --- | --- | --- | --- |
| **Frequency Percent Row Pct Col Pct** | **Allele 2** | **Allele 3** | **Allele 4** | **Allele 5** | **Alleles >5** | **Total** |
| **West** | 12 12.63 21.82 52.17 | 5 5.26 9.09 62.50 | 17 17.89 30.91 51.52 | 7 7.37 12.73 53.85 | 14 14.74 25.45 77.78 | 55 57.89 |
| **East** | 11 11.58 27.50 47.83 | 3 3.16 7.50 37.50 | 16 16.84 40.00 48.48 | 6 6.32 15.00 46.15 | 4 4.21 10.00 22.22 | 40 42.11 |
| **Total** | 23 24.21 | 8 8.42 | 33 34.74 | 13 13.68 | 18 18.95 | 95 100.00 |

| **Cross-tabulation of Locus-6 by region** | | | | |
| --- | --- | --- | --- | --- |
| **Frequency Percent Row Pct Col Pct** | **Allele “M”** | **Allele 2** | **Alleles > 2** | **Total** |
| **West** | 6 6.32 10.91 37.50 | 45 47.37 81.82 63.38 | 4 4.21 7.27 50.00 | 55 57.89 |
| **East** | 10 10.53 25.00 62.50 | 26 27.37 65.00 36.62 | 4 4.21 10.00 50.00 | 40 42.11 |
| **Total** | 16 16.84 | 71 74.74 | 8 8.42 | 95 100.00 |

| **Cross-tabulation of Locus-7 by region** | | | | | |
| --- | --- | --- | --- | --- | --- |
| **Frequency Percent Row Pct Col Pct** | **Alleles 3, and 4** | **Alleles 5, 6 and 7** | **Alleles 8, 9 and 10** | **Alleles >10** | **Total** |
| **West** | 8 8.42 14.55 53.33 | 16 16.84 29.09 53.33 | 22 23.16 40.00 61.11 | 9 9.47 16.36 64.29 | 55 57.89 |
| **East** | 7 7.37 17.50 46.67 | 14 14.74 35.00 46.67 | 14 14.74 35.00 38.89 | 5 5.26 12.50 35.71 | 40 42.11 |
| **Total** | 15 15.79 | 30 31.58 | 36 37.89 | 14 14.74 | 95 100.00 |
